# Supplementary material for: Interplay between human STING genotype and bacterial NADase activity regulates inter-individual disease variability
Source: Nat Commun. 2023 Jul 6;14:4008. doi: 10.1038/s41467-023-39771-0 (PMC10326033; doi:10.1038/s41467-023-39771-0)
Supplement: Supplementary file 1 — Supplementary Information [file 41467_2023_39771_MOESM1_ESM.pdf]

Supplementary information for:

Interplay between human STING genotype and bacterial NADase activity regulates interindividual disease variability

Elin Movert *et al.*

Contents:

- Supplementary Fig. 1 – 10, including figure legends
- Supplementary Table 1, including legend
- Legend for Supplementary Data 1 (separate XLSX-file)
- Supplementary references
- Source Data for Fig. 1l

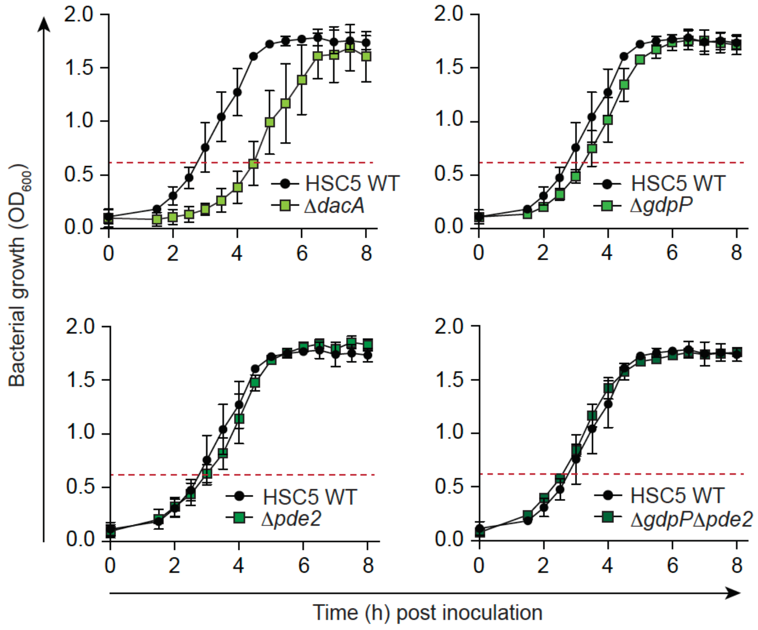

Supplementary Fig. 1 | *S. pyogenes* deletion mutants affected in c-di-AMP metabolism exhibit similar growth rates as wild type bacteria in exponential phase. Overnight cultures were reinoculated into prewarmed THY broth and bacterial growth was assessed by measuring OD_{600} at different timepoints post inoculation, as indicated. Results (mean ± SD; *n*=3) representative of three independent experiments. Red dotted line indicates the OD_{600} at which bacteria were harvested for macrophage infections.

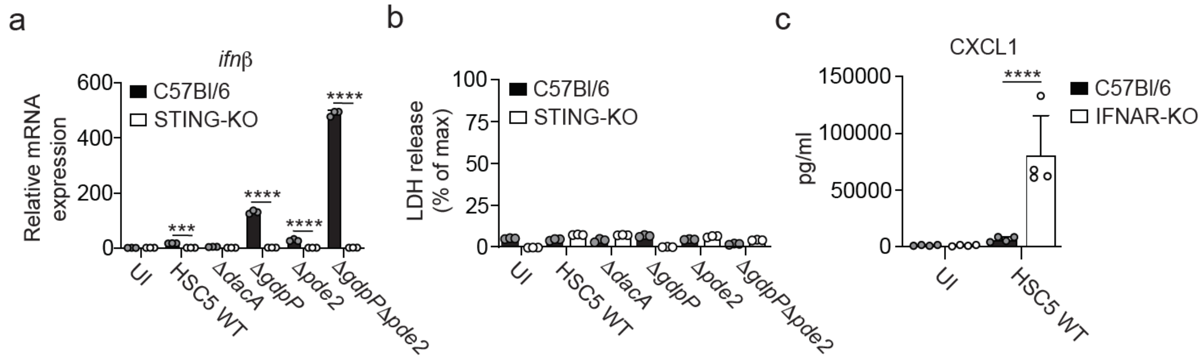

Supplementary Fig. 2 | Type I IFN induction by bacteria-derived c-di-AMP is dependent on STING, and type I IFN signaling inhibits the production of CXCL1. a, RTqPCR analysis of *ifnβ* expression in C57Bl/6 wild type or STING-KO macrophages infected with HSC5 wild type (HSC5 WT), its isogenic deletion mutants (Δ*dacA,* Δ*gdpP,* Δ*pde2* and Δ*gdpP*Δ*pde2*) or uninfected (UI) controls, as indicated. **b**, Analysis of LDH release as a measure of cytotoxicity at 4 hours post infection, as indicated. (**a-b**) Results (mean and SD; *n*=3) representative of two independent experiments. **c**, Analysis of CXCL1 secreted from C57Bl/6 or IFNAR-KO macrophages infected with HSC5 WT or uninfected (UI). Results (mean and SD; *n*=4) representative of two independent experiments. 1-way ANOVA with Dunnett's test. **p*<0.05, ***p*<0.01, ****p*<0.001, *****p*<0.0001.

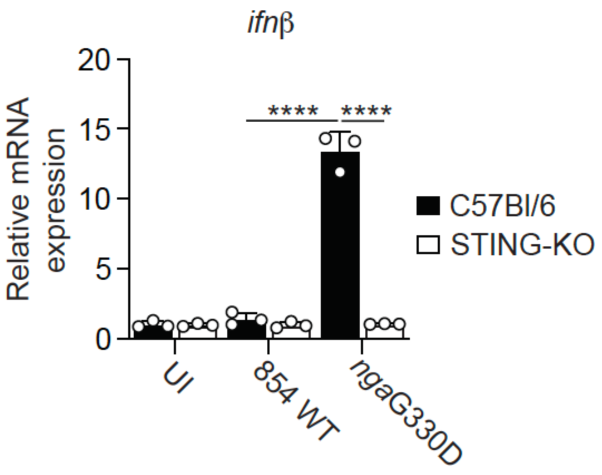

Supplementary Fig. 3 | The increased type I IFN production in *nga*G330D infected macrophages is dependent on STING. RTqPCR analysis of *ifnβ* expression in C57Bl/6 wild type or STING-KO macrophages infected with M1 (854) WT, the *nga*G330D isogenic mutant, or uninfected (UI) control. Results (mean and SD; n=3) representative of three independent experiments. 1-way ANOVA with Dunnett's test. *p<0.05, **p<0.01, ***p<0.001, ****p<0.0001.

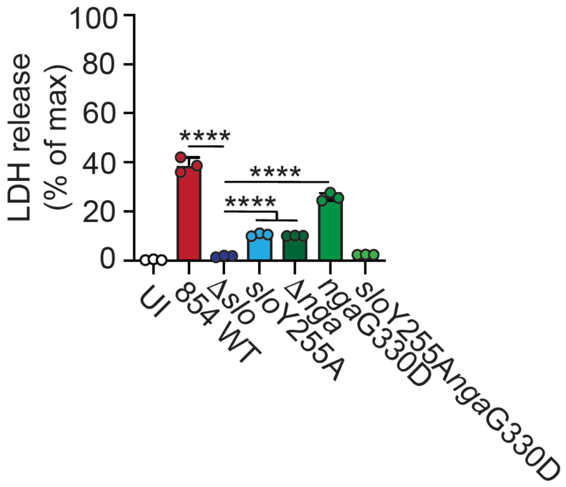

Supplementary Fig. 4 | Cell death is not the cause of differential type I IFN production in macrophages infected with *S. pyogenes* mutants affected in NADase and/or SLO function. Cytotoxicity assessment by relative LDH release at 4 hpi in C57Bl/6 macrophages infected with M1 (854) WT, the isogenic mutants (Δ*slo*, sloY255A, Δ*nga*, *nga*G330D and *slo*Y255A*nga*G330D) or uninfected (UI) control. Results (mean and SD; *n*=3) representative of three independent experiments. 1-way ANOVA with Dunnett's test. *$p<0.05$, **$p<0.01$, ***$p<0.001$, ****$p<0.0001$.

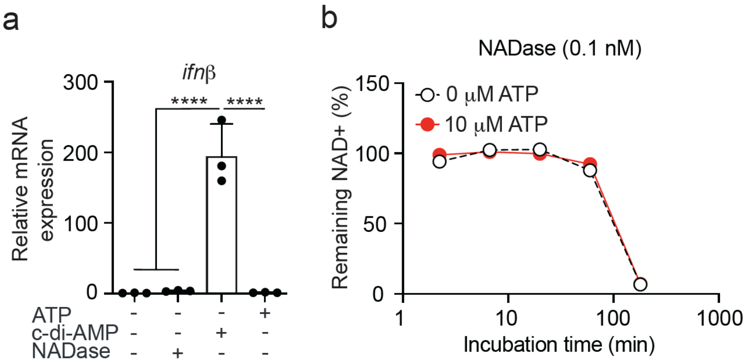

Supplementary Fig. 5 | Addition of ATP alone does not drive *ifnβ* expression in macrophages or inhibit the enzymatic activity of *S. pyogenes* NADase. a, RTqPCR analysis of *ifnβ* expression in transiently permeabilized (10 μg/ml digitonin) C57Bl/6 macrophages treated with ATP (1 mM), c-di-AMP (7.5 μM), recombinant NADase (0.5 μM), or left untreated (UT), as indicated. Analysis was performed 2,5 hours post treatment. Results (mean and SD; *n*=3) are representative of three independent experiments. 1-way ANOVA with Dunnett's test. *p<0.05, **p<0.01, ***p<0.001, ****p<0.0001. **b-c**, Enzymatic activity of purified recombinant NADase as assessed by NAD degradation over time ± ATP, as indicated. Results are representative of at least three independent experiments. 2-way ANOVA with Tukey's test. *p<0.05, **p<0.01, ***p<0.001, ****p<0.0001.

a

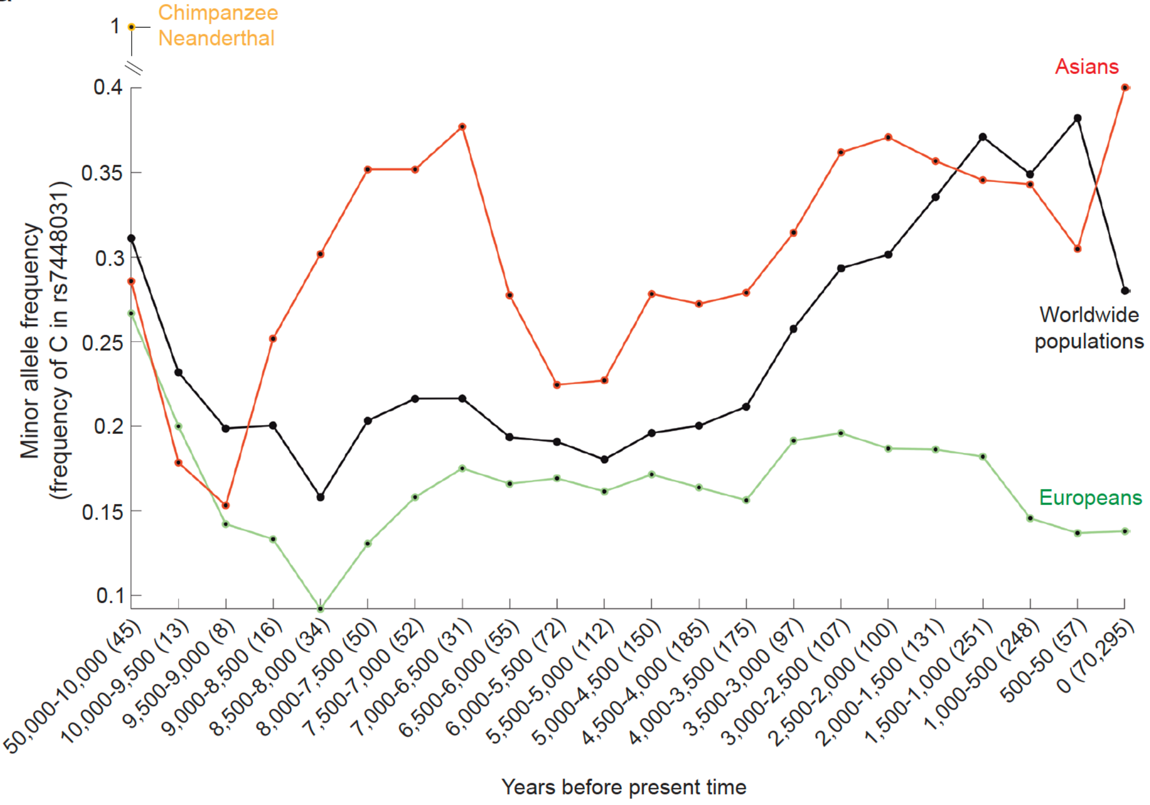

Chimpanzee
Neanderthal
Asians
Worldwide populations
Europeans
Minor allele frequency (frequency of C in rs7448031)
1
0.4
0.35
0.3
0.25
0.2
0.15
0.1
50,000-10,000 (45)
10,000-9,500 (13)
9,500-9,000 (8)
9,000-8,500 (16)
8,500-8,000 (34)
8,000-7,500 (50)
7,500-7,000 (52)
7,000-6,500 (31)
6,500-6,000 (55)
6,000-5,500 (72)
5,500-5,000 (112)
5,000-4,500 (150)
4,500-4,000 (185)
4,000-3,500 (175)
3,500-3,000 (97)
3,000-2,500 (107)
2,500-2,000 (100)
2,000-1,500 (131)
1,500-1,000 (251)
1,000-500 (248)
500-50 (57)
0 (70,295)
Years before present time

b

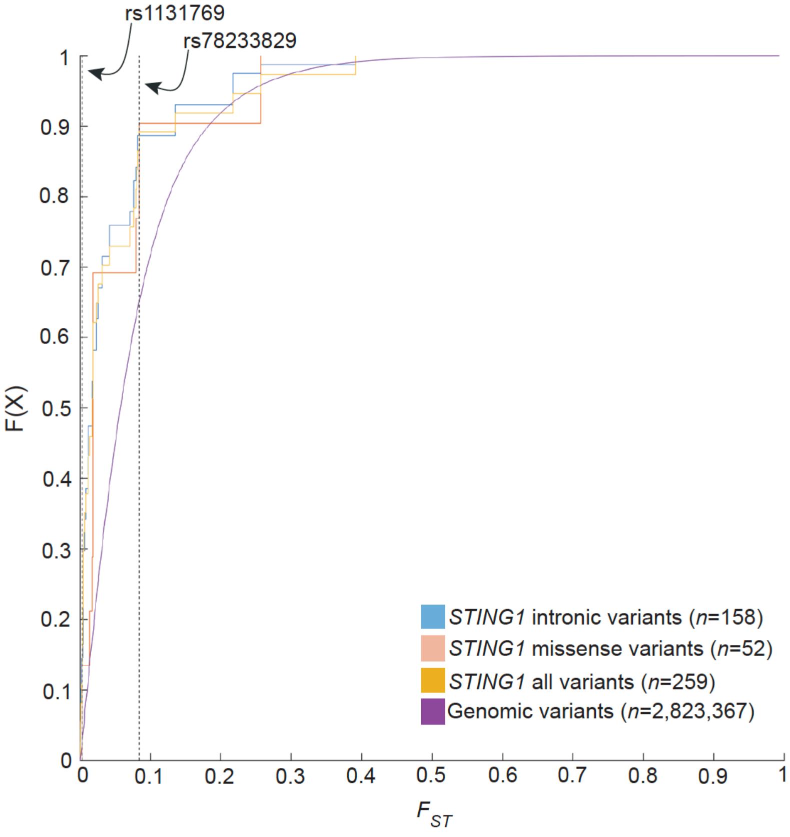

rs1131769
rs78233829
F(X)
1
0.9
0.8
0.7
0.6
0.5
0.4
0.3
0.2
0.1
0
0.1
0.2
0.3
0.4
0.5
0.6
0.7
0.8
0.9
1
STING1 intronic variants (n=158)
STING1 missense variants (n=52)
STING1 all variants (n=259)
Genomic variants (n=2,823,367)
F_{ST}

Supplementary Fig. 6 | The evolutionary history of STING in humans. a, The SNPs responsible for STING-G230A and STING-R232H were not present in ancient DNA datasets. However, rs78233829 (C/G; G230A) is in linkage disequilibrium (r^2 and D' >0.95) with rs7448031 (T/C), a transcription binding site variant present in the datasets. The allele frequency of rs78233829's tag-SNP (rs7448031 C allele; minor allele) is shown for Asians, Europeans and worldwide populations, as indicated. All 8 Denisovans and Neanderthals were homozygotes for the minor allele, similarly to chimpanzee, whereas 11/12 early humans (45,000 to 32,000 years ago) were homozygotes for the major allele. The minor allele is first detected in humans only ~35,000 years ago. Remarkably, its allele frequency decreased in all humans until the Neolithic revolution, and subsequently exhibited opposite patterns in Asians and Europeans with an increase in the former while remaining low in the latter. Modern allele frequencies were obtained from gnomAD for Europeans (Middle Easterns and Europeans), Asians (East Asians), Latino, and Africans with the global frequency calculated as the arithmetic mean of the allele frequencies of these four groups. Total sample sizes analyzed are shown within parenthesis in the *x*-axis. **b**, Analysis of the cumulative distribution function of the genomic and STING continental F_{ST}. The genomic distribution of F_{ST} for ~2.8M SNPs is shown alongside the F_{ST} distribution of STING variants calculated for Ensembl/GENCODE transcripts. F_{ST} of two STING SNPs are shown (dashed lines), as indicated. The average continental F_{ST} of STING variants is lower than the genomic F_{ST}[1]. Yet, rs78233829's F_{ST} (0.084) is higher than the genomic median and ~90% of STING variants, suggestive of local adaptation.

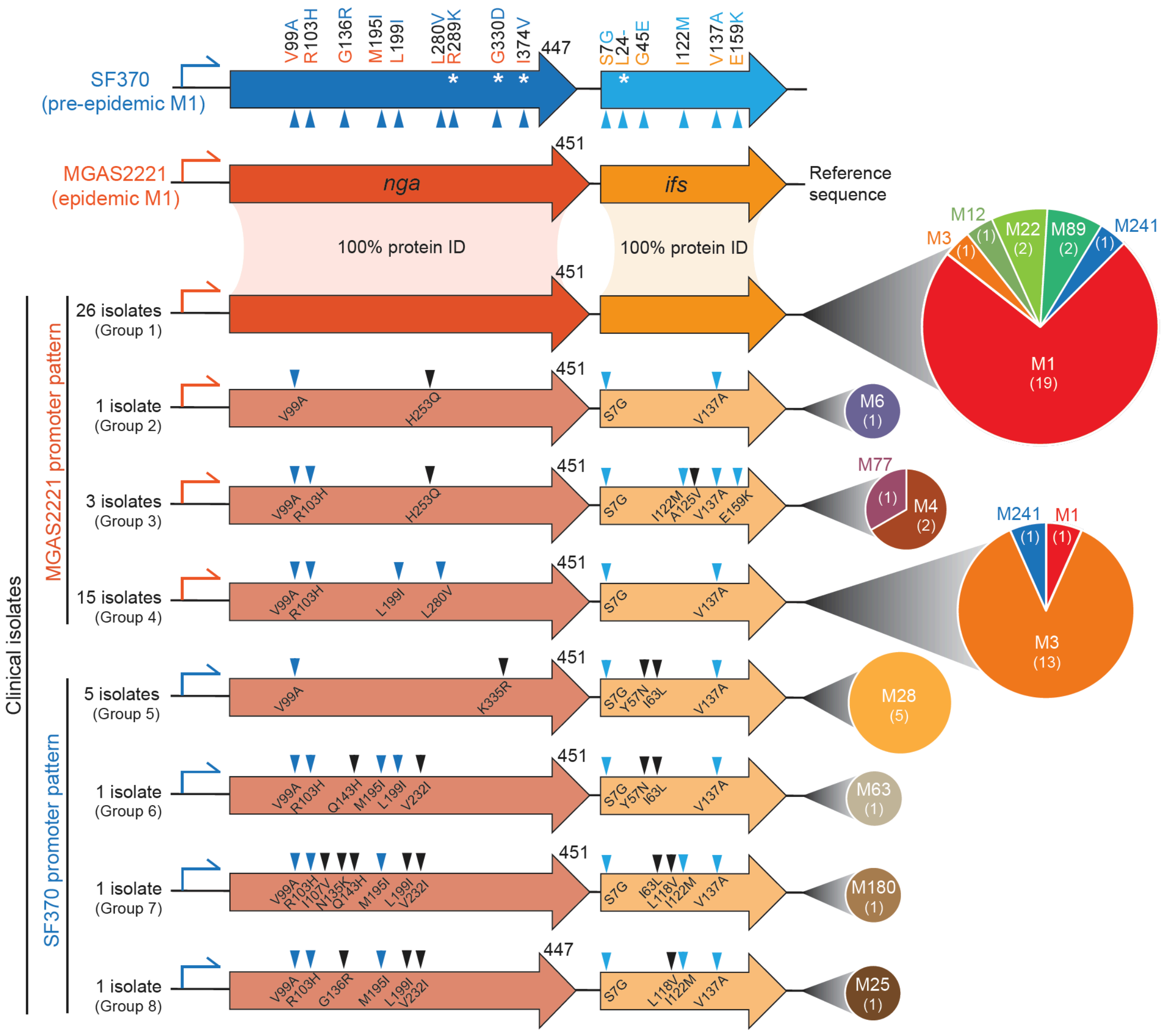

Supplementary Fig. 7 | *nga*-region sequence analysis of the 53 *S. pyogenes* isolates from NSTI patient. Schematic overview of the polymorphic residues in *nga* and *ifs* of our clinical isolates and pre-epidemic M1 SF370, compared to the epidemic M1 strain MGAS2221. Specific amino acid substitutions known to render NADase less enzymatically active are indicated with white asterisks (in SF370)[2], and the nonsense mutation L24- that produces a truncated (nonfunctional) IFS is similarly indicated[3]. The total NADase protein sequence length for the analyzed strains is either 447 or 451, as indicated. Previously described polymorphisms are indicated with blue arrowheads, and new polymorphisms are indicated with black arrowheads. The two *nga* promotor pattern variants, associated with epidemic and pre-

epidemic M1 strains, respectively[4], are indicated for each group. For each group the distribution of *emm* types is shown in a pie chart with the number of isolates of each *emm* type indicated within parenthesis.

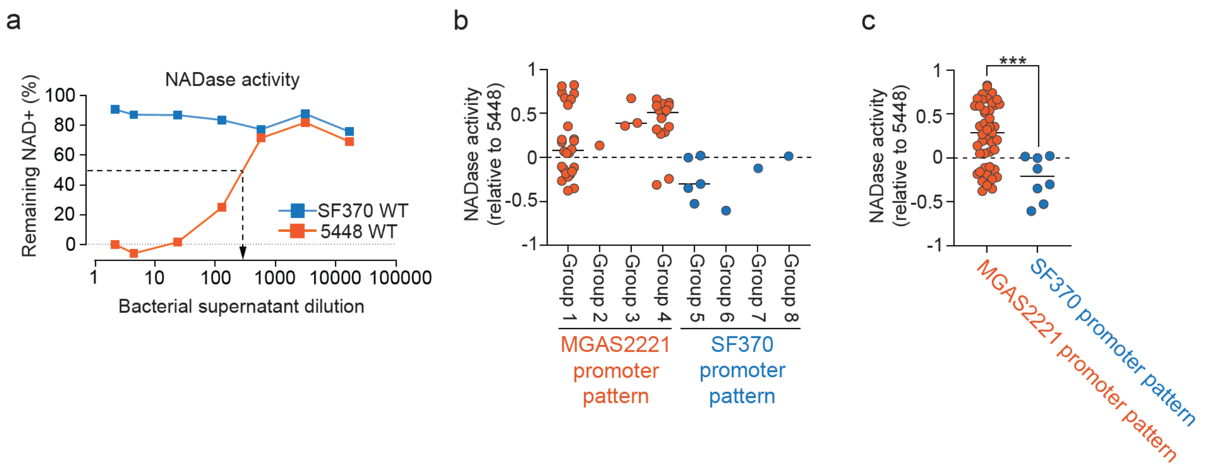

Supplementary Fig. 8 | All analyzed *S. pyogenes* NSTI patient isolates express an enzymatically active NADase. a, The pre-epidemic M1 SF370 strain and the epidemic reference strain 5448 – which exhibits 100% *nga*-region sequence identity to the epidemic reference strain MGAS2221 (**Supplementary Fig. 7**) – were included as controls in all experiments to allow comparative analysis of NADase activity in overnight cultures for each of the 53 *S. pyogenes* isolates from NSTI patients. For each experiment, data was normalized to the dilution value corresponding to 50% of remaining NAD for 5448 (indicated by the arrow), and log transformed. **b**, Relative NADase activity in the 53 NSTI patient isolates divided into groups based on *nga*-region sequence, or **c**, divided based on *nga* promotor pattern (see **Supplementary Fig. 7** for definition of groups). Results are based on three independent experiments. (**c**) Two-sided unpaired *t*-test. **p*<0.05, ***p*<0.01, ****p*<0.001, *****p*<0.0001.

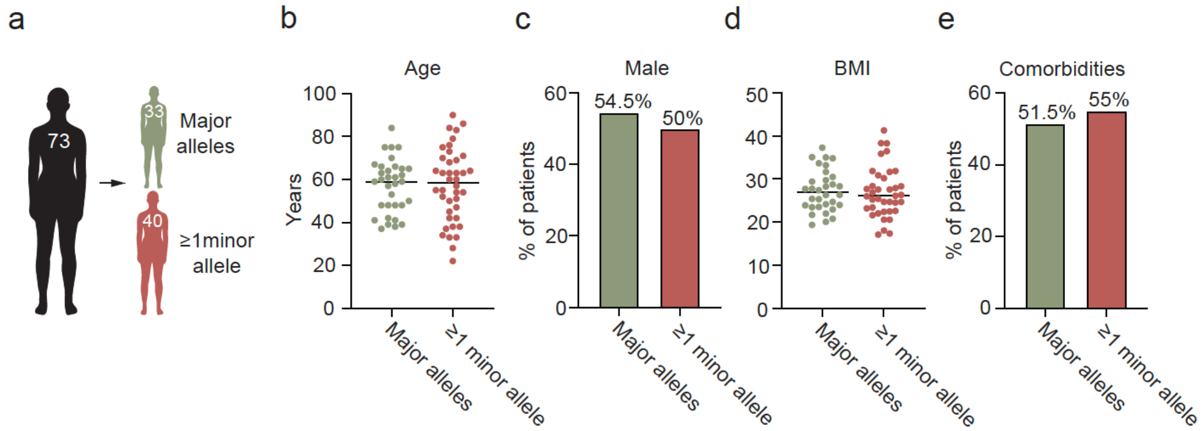

Supplementary Fig. 9 | STING genotype within the NSTI patient cohort shows an even distribution for age, sex and risk factors. a, Sequencing of the STING-encoding *STING1* gene demonstrated that 33 of the 73 NSTI patients were homozygous for the major allele and 40 were at least heterozygotes for one minor allele. These two groups exhibited no significant differences in (**b**) age, (**c**) sex, (**d**) BMI or (**e**) comorbidities as previously defined[5].

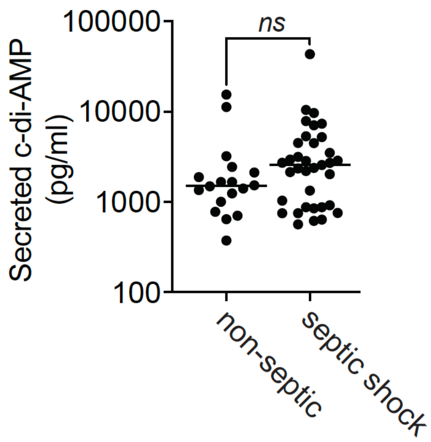

Supplementary Fig. 10 | The amount of c-di-AMP secreted from *S. pyogenes* strains isolated from NSTI patient does not correlate with the development of septic shock. Analysis of c-di-AMP concentration in culture supernatants (*i.e.* secreted c-di-AMP) from the 53 *S. pyogenes* NSTI patient isolates grouped based on whether or not the patients developed septic shock. Results are based on three independent experiments. Two-sided unpaired *t*-test, non-significant (*ns*).

Supplementary Table 1 | Materials and reagents

Streptococcus pyogenes strains	Source	Reference
SF370 (M1, ATCC; strain reference 700294)	Gunnar Lindahl, Lund University	[6]
5448 (M1T1)	Nina van Sorge, Utrecht University	[7]
854 (M1T1)	Michael R Wessels, Harvard Medical School	[8]
854 Δ*nga*	Michael R Wessels, Harvard Medical School	[9]
854 *nga*G330D	Michael R Wessels, Harvard Medical School	[9]
854 Δ*slo*	Michael R Wessels, Harvard Medical School	[9]
854 *slo*Y255A	Michael R Wessels, Harvard Medical School	[9]
854 *slo*Y255A*nga*G330D	Michael R Wessels, Harvard Medical School	[9]
950771 (M3)	Michael R Wessels, Harvard Medical School	[10]
950771Sm Δ*slo*	Michael R Wessels, Harvard Medical School	[11]
HSC5 (M14)	Kyu Hong Cho, Indiana State University	[12]
HSC5 Δ*gdpP*	Kyu Hong Cho, Indiana State University	[13]
HSC5 Δ*dacA*	Kyu Hong Cho, Indiana State University	[14]
HSC5 Δ*pde2*	Kyu Hong Cho, Indiana State University	[14]
HSC5 Δ*gdpP*Δ*pde2*	Kyu Hong Cho, Indiana State University	[14]
Isolates from 53 NSTI patients included in the INFECT project (EU-FP7-HEALTH)*	Anna Norrby-Teglund, the Karolinska Institute	[5]
**Recombinant proteins**	**Source**	**Reference**
NADase	Michael R Wessels, Harvard Medical School	[15]
NADase G330D (G330D)	Michael R Wessels, Harvard Medical School	[15]
**Antibodies**	**Source**	**Catalog number**
Polyclonal rabbit anti-mouse STAT1	Cell Signaling Technologies	Cat# 9172S
Monoclonal rabbit anti-mouse phosphoSTAT1 (p-Tyr701)	Cell Signaling Technologies	Cat# 9167S
Polyclonal goat anti-rabbit IgG conjugated with Horseradish Peroxidase	Jackson ImmunoResearch	Cat# 111-036-003
**Commercial assays**	**Source**	**Catalog number**
Mouse IFN-beta DuoSet ELISA	R&D Systems	Cat# DY8234
Mouse CXCL1/KC DuoSet ELISA	R&D Systems	Cat# DY453
TNF alpha Mouse Uncoated ELISA Kit	Invitrogen	Cat# 88-7324-88
Cyclic di-AMP ELISA Kit	Cayman	Cat# 501960
Cytotoxicity Assay (LDH release)	Promega	Cat# G1780
Luminescent ATP Detection Assay Kit	Abcam	Cat# ab113849
RNeasy mini kit	Qiagen	Cat# 74104
GoScript Reverse transcription system	Promega	Cat# A5003
SSoFast EvaGreen qPCR supermix	Biorad	Cat# 1725204
QIAamp DNA Blood Maxi Kit	Qiagen	Cat# 51194
TrueStart Hot Start Taq DNA polymerase	Thermo Scientific	Cat# 10540081
**Chemicals and molecules**	**Source**	**Catalog number**
Nonidet P-40	Sigma-Aldrich	Cat# I8896
cOmplete™, EDTA-free Protease Inhibitor Cocktail	Roche	Cat# 11873580001
PhosSTOP, phosphatase inhibitor cocktail	Roche	Cat# 4906845001
Digitonin	Sigma-Aldrich	Cat# D141
β-Nicotinamide adenine dinucleotide hydrate (NAD)	Sigma-Aldrich	Cat# N7004 and N1636
c-di-AMP	InvivoGen	Cat# tlrl-nacda
c-di-GMP	InvivoGen	Cat# tlrl-nacdg
pI:C	InvivoGen	Cat# trlr-pIc

pTEC15 plasmid	Addgene	Cat# 30174
LPS	Sigma-Aldrich	Cat# L2630
ATP	Thermo Fischer Scientific	Cat# R0441
Phosphodiesterase I from *Crotalus adamanteus* venom	Sigma-Aldrich	Cat# P3134

Primers	Sequence (5´- 3')
Mouse *reep5* (forward)	GCCATCGAGAGTCCCAACAA
Mouse *reep5* (reverse)	GCATCTCAGCCCCATTAGC
Mouse *ifnb* (forward)	ATGAGTGGTGGTTGCAGGC
Mouse *ifnb* (reverse)	TGACCTTTCAAATGCAGTAGATTCA
Mouse *tnfa* (forward)	AGGGTCTGGGCCATAGAACT
Mouse *tnfa* (reverse)	CCACCACGCTCTTCTGTCTAC
Human *STING1* sequencing primer (forward)	GGCTGTATATTCTCCTCCCATTG
Human *STING1* sequencing primer (reverse)	AGCTTGTAGTAAGTGCTCGATAAA

Equipment	Source
Trans-blot Turbo transfer system	Biorad
ChemiDoc Imaging systems	Biorad
Ultrospec 10 cell density meter (600 nm)	Biochrom
ND-1000 Spectrophotometer Nanodrop®	Saveen Werner
SpektraMAX i3x plate reader	Molecular Devices
CFX384™ Real-time System C1000 Touch™ Thermal Cycler and CFX Maestro software	Biorad

* Whole genome sequences stored in the European Nucleotide Archive, BioProject PRJNA524111.

Supplementary Data 1 | List of the 1999 ancient DNA samples used to genotype rs78233829's tag-SNP rs7448031 (separate XLSX-file).

Supplementary references

1 Elhaik, E. Empirical distributions of F(ST) from large-scale human polymorphism data. *PLoS One* **7**, e49837 (2012).

2 Chandrasekaran, S., Ghosh, J., Port, G. C., Koh, E. I. & Caparon, M. G. Analysis of polymorphic residues reveals distinct enzymatic and cytotoxic activities of the Streptococcus pyogenes NAD+ glycohydrolase. *J Biol Chem* **288**, 20064-20075 (2013).

3 Meehl, M. A., Pinkner, J. S., Anderson, P. J., Hultgren, S. J. & Caparon, M. G. A novel endogenous inhibitor of the secreted streptococcal NAD-glycohydrolase. *PLoS Pathog* **1**, e35 (2005).

4 Zhu, L. *et al.* A molecular trigger for intercontinental epidemics of group A Streptococcus. *J Clin Invest* **125**, 3545-3559 (2015).

5 Bruun, T. *et al.* Risk Factors and Predictors of Mortality in Streptococcal Necrotizing Soft-tissue Infections: A Multicenter Prospective Study. *Clin Infect Dis* **72**, 293-300 (2021).

6 Ferretti, J. J. *et al.* Complete genome sequence of an M1 strain of Streptococcus pyogenes. *Proc Natl Acad Sci U S A* **98**, 4658-4663 (2001).

7 Kansal, R. G., McGeer, A., Low, D. E., Norrby-Teglund, A. & Kotb, M. Inverse relation between disease severity and expression of the streptococcal cysteine protease, SpeB, among clonal M1T1 isolates recovered from invasive group A streptococcal infection cases. *Infect Immun* **68**, 6362-6369 (2000).
8 Gryllos, I. *et al.* Induction of group A Streptococcus virulence by a human antimicrobial peptide. *Proc Natl Acad Sci U S A* **105**, 16755-16760 (2008).
9 Bastiat-Sempe, B., Love, J. F., Lomayesva, N. & Wessels, M. R. Streptolysin O and NAD-glycohydrolase prevent phagolysosome acidification and promote group A Streptococcus survival in macrophages. *mBio* **5**, e01690-01614 (2014).
10 Ashbaugh, C. D., Warren, H. B., Carey, V. J. & Wessels, M. R. Molecular analysis of the role of the group A streptococcal cysteine protease, hyaluronic acid capsule, and M protein in a murine model of human invasive soft-tissue infection. *J Clin Invest* **102**, 550-560 (1998).
11 Bricker, A. L., Cywes, C., Ashbaugh, C. D. & Wessels, M. R. NAD+-glycohydrolase acts as an intracellular toxin to enhance the extracellular survival of group A streptococci. *Mol Microbiol* **44**, 257-269 (2002).
12 Hanski, E. & Caparon, M. Protein F, a fibronectin-binding protein, is an adhesin of the group A streptococcus Streptococcus pyogenes. *Proc Natl Acad Sci U S A* **89**, 6172-6176 (1992).
13 Cho, K. H. & Kang, S. O. Streptococcus pyogenes c-di-AMP Phosphodiesterase, GdpP, Influences SpeB Processing and Virulence. *Plos One* **8**, e69425 (2013).
14 Fahmi, T., Faozia, S., Port, G. C. & Cho, K. H. The Second Messenger c-di-AMP Regulates Diverse Cellular Pathways Involved in Stress Response, Biofilm Formation, Cell Wall Homeostasis, SpeB Expression, and Virulence in Streptococcus pyogenes. *Infect Immun* **87**, doi:10.1128/IAI.00147-19 (2019).
15 Sharma, O., O'Seaghdha, M., Velarde, J. J. & Wessels, M. R. NAD+-Glycohydrolase Promotes Intracellular Survival of Group A Streptococcus. *PLoS Pathog* **12**, e1005468 (2016).

Source Data

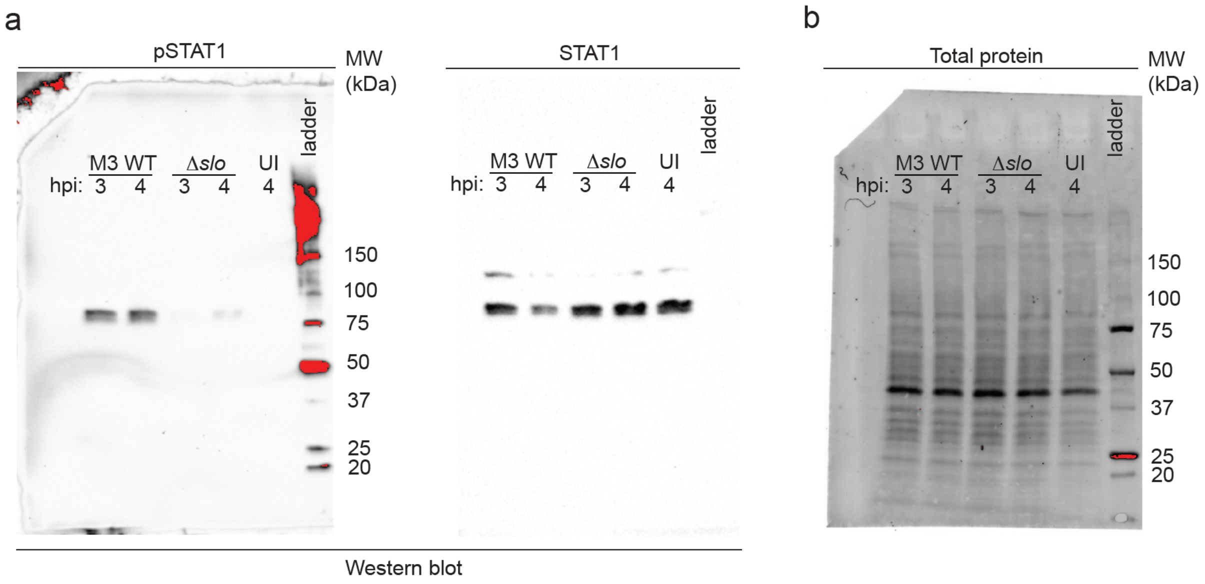

Source data for the analysis presented in Fig. 1l. a, Uncropped and unprocessed Western blot scans of STAT1 activation analysis using antibodies specific for phosphorylated STAT1 (pSTAT) and total STAT1, as indicated. The molecular weight ladder is visible in the pSTAT1 blot. **b,** Uncropped and unprocessed scan of total protein content on membrane used for Western blot analysis in **a**. Molecular weight marker (ladder) in kDa, as indicated.
