# Supplementary material for: Interplay between human STING genotype and bacterial NADase activity regulates inter-individual disease variability
Source: Nat Commun. 2023 Jul 6;14:4008. doi: 10.1038/s41467-023-39771-0 (PMC10326033; doi:10.1038/s41467-023-39771-0)
Supplement: Supplementary file 3 — Description of Additional Supplementary Files [file 41467_2023_39771_MOESM3_ESM.pdf]

Description of Additional Supplementary Files

Supplementary Data 1. List of the 1999 ancient DNA samples used to genotype rs78233829's tag-SNP rs7448031 (separate XLSX-file).
