# Supplementary material for: Interplay between human STING genotype and bacterial NADase activity regulates inter-individual disease variability
Source: Nat Commun. 2023 Jul 6;14:4008. doi: 10.1038/s41467-023-39771-0 (PMC10326033; doi:10.1038/s41467-023-39771-0)
Supplement: Supplementary file 5 — Reporting Summary [file 41467_2023_39771_MOESM5_ESM.pdf]

Corresponding author(s): Fredric Carlsson

Last updated by author(s): May 30, 2023

Reporting Summary

Nature Portfolio wishes to improve the reproducibility of the work that we publish. This form provides structure for consistency and transparency in reporting. For further information on Nature Portfolio policies, see our Editorial Policies and the Editorial Policy Checklist.

Statistics

For all statistical analyses, confirm that the following items are present in the figure legend, table legend, main text, or Methods section.

n/a	Confirmed	
☐	☒	The exact sample size (*n*) for each experimental group/condition, given as a discrete number and unit of measurement
☐	☒	A statement on whether measurements were taken from distinct samples or whether the same sample was measured repeatedly
☐	☒	The statistical test(s) used AND whether they are one- or two-sided<br>*Only common tests should be described solely by name; describe more complex techniques in the Methods section.*
☐	☒	A description of all covariates tested
☐	☒	A description of any assumptions or corrections, such as tests of normality and adjustment for multiple comparisons
☐	☒	A full description of the statistical parameters including central tendency (e.g. means) or other basic estimates (e.g. regression coefficient) AND variation (e.g. standard deviation) or associated estimates of uncertainty (e.g. confidence intervals)
☐	☒	For null hypothesis testing, the test statistic (e.g. *F*, *t*, *r*) with confidence intervals, effect sizes, degrees of freedom and *P* value noted<br>*Give P values as exact values whenever suitable.*
☒	☐	For Bayesian analysis, information on the choice of priors and Markov chain Monte Carlo settings
☒	☐	For hierarchical and complex designs, identification of the appropriate level for tests and full reporting of outcomes
☒	☐	Estimates of effect sizes (e.g. Cohen's *d*, Pearson's *r*), indicating how they were calculated

Our web collection on statistics for biologists contains articles on many of the points above.

Software and code

Policy information about availability of computer code

Data collection	CFX Maestro 2.0 (Biorad) for real-time qPCR data collection.<br>ChemiDoc Imaging systems (Biorad) for Western blot data collection.<br>SpektraMAX i3x plate reader (Molecular Devises) for ELISA data collection.
Data analysis	Prismv8 was used to generate graphs and perform statistical analyses.<br>CFX384™ Real-time System C1000 Touch™ Thermal Cycler and CFX Maestro software.

For manuscripts utilizing custom algorithms or software that are central to the research but not yet described in published literature, software must be made available to editors and reviewers. We strongly encourage code deposition in a community repository (e.g. GitHub). See the Nature Portfolio guidelines for submitting code & software for further information.

Data

Policy information about availability of data

All manuscripts must include a data availability statement. This statement should provide the following information, where applicable:

- Accession codes, unique identifiers, or web links for publicly available datasets
- A description of any restrictions on data availability
- For clinical datasets or third party data, please ensure that the statement adheres to our policy

The authors declare that the data supporting the findings of this study are available within the paper and its supplementary files. The ancient genomes are available

at the Allen Ancient DNA Resource (https://reich.hms.harvard.edu/allen-ancient-dna-resource-aadr-downloadable-genotypes-present-day-and-ancient-dna-data), and the complete bacterial genomes of the clinical strains are available at the European Nucleotide Archive (https://www.ebi.ac.uk/ena/), BioProject PRJNA524111.

Human research participants

Policy information about studies involving human research participants and Sex and Gender in Research.

Reporting on sex and gender	Reported in Supplementary Fig. 10C, and described in detail in:<br><br>Madsen, M. B. et al. Patient's characteristics and outcomes in necrotising soft-tissue infections: results from a Scandinavian, multicentre, prospective cohort study. Intensive Care Med 45, 1241-1251 (2019).<br><br>Madsen, B.B. et al. Necrotizing soft tissue infections - a multicenter, prospective observational study (INFECT): protocol and statistical analysis plan. Acta Anaesthesiological Scandinavia 62, 272-279 (2018).
Population characteristics	Reported in Supplementary Fig. 10, and described in detail in:<br><br>Madsen, M. B. et al. Patient's characteristics and outcomes in necrotising soft-tissue infections: results from a Scandinavian, multicentre, prospective cohort study. Intensive Care Med 45, 1241-1251 (2019).<br><br>Madsen, B.B. et al. Necrotizing soft tissue infections - a multicenter, prospective observational study (INFECT): protocol and statistical analysis plan. Acta Anaesthesiological Scandinavia 62, 272-279 (2018).
Recruitment	Described in:<br><br>Madsen, M. B. et al. Patient's characteristics and outcomes in necrotising soft-tissue infections: results from a Scandinavian, multicentre, prospective cohort study. Intensive Care Med 45, 1241-1251 (2019).<br><br>Madsen, B.B. et al. Necrotizing soft tissue infections - a multicenter, prospective observational study (INFECT): protocol and statistical analysis plan. Acta Anaesthesiological Scandinavia 62, 272-279 (2018).
Ethics oversight	Work involving human samples was approved by the Danish Ethical Committee (1211709), the Swedish Ethical Committee (Dnr: 930-12) and the Regional Committee for Ethics in Medical Research (2012/2227/REK VEST) in Western Norway.

Note that full information on the approval of the study protocol must also be provided in the manuscript.

Field-specific reporting

Please select the one below that is the best fit for your research. If you are not sure, read the appropriate sections before making your selection.

- [x] Life sciences
- [ ] Behavioural & social sciences
- [ ] Ecological, evolutionary & environmental sciences

For a reference copy of the document with all sections, see nature.com/documents/nr-reporting-summary-flat.pdf

Life sciences study design

All studies must disclose on these points even when the disclosure is negative.

Sample size	The sample size was not formally calculated but based on prior knowledge and publication (Movert E. PLos Pathogens 2018).
Data exclusions	No data were excluded from the analyses.
Replication	Experiments were repeated as indicated in the figure legends and were always reproducible.
Randomization	Randomization was not performed because no subjective measurements were used.
Blinding	Blinding was not performed because no subjective measurements were used.

Reporting for specific materials, systems and methods

We require information from authors about some types of materials, experimental systems and methods used in many studies. Here, indicate whether each material, system or method listed is relevant to your study. If you are not sure if a list item applies to your research, read the appropriate section before selecting a response.

Materials & experimental systems

n/a	Involved in the study	
	x	Antibodies
x		Eukaryotic cell lines
x		Palaeontology and archaeology
	x	Animals and other organisms
	x	Clinical data
x		Dual use research of concern

Methods

n/a	Involved in the study	
x		ChIP-seq
x		Flow cytometry
x		MRI-based neuroimaging

Antibodies

Antibodies used

Antibody (supplier name, #catalog number):

Polyclonal rabbit anti-mouse STAT1 (Cell Signaling Technologies, Cat# 9172S)
Monoclonal rabbit anti-mouse phosphoSTAT1 (p-Tyr701) (Cell Signaling Technologies, Cat# 9167S)
Polyclonal goat anti-rabbit IgG conjugated with Horseradish Peroxidase (Jackson ImmunoResearch, Cat# 111-036-003)

Validation

The polyclonal rabbit anti-mouse STAT1 Abs (Cell Signaling Technologies, Cat# 9172S) recognize mouse STAT1 and is validated for Western blot, as indicated at the manufacturer's website.
The monoclonal rabbit anti-mouse phosphoSTAT1 Ab (p-Tyr701) (Cell Signaling Technologies, Cat# 9167S) recognizes Tyr701-phosphorylated mouse STAT1 and is validated for Western blot, as indicated at the manufacturer's website.
The polyclonal goat anti-rabbit IgG conjugated with Horseradish Peroxidase (Jackson ImmunoResearch, Cat# 111-036-003) is specific for rabbig IgG (H+L) and validated for Western blot, as indicated at the manufacturer's website.

Animals and other research organisms

Policy information about studies involving animals; ARRIVE guidelines recommended for reporting animal research, and Sex and Gender in Research

Laboratory animals

Wild type C57Bl/6JRj, Ifnar1-/- (IFNAR-KO) and Tmem173gt (STING-KO) mice. Wild type and IFNAR-KO mice were bred and maintained at the Biology Department animal facility. Bone marrow was isolated from mice at 8 weeks of age. Bone marrow from STING-KO animals was isolated and kindly provided by Russell Vance (UC Berkeley, USA).

Wild animals

The study did not involve wild animals.

Reporting on sex

Both male and female mice were used to generate bone marrow-derived macrophages.

Field-collected samples

The study did not involve samples collected from the field.

Ethics oversight

All animal experimentations were approved by the Malmö/Lund Ethical Board for Animal Research, Sweden (permit number 5.8.18-07342/2017 and 5.8.18-08454/2020)

Note that full information on the approval of the study protocol must also be provided in the manuscript.

Clinical data

Policy information about clinical studies
All manuscripts should comply with the ICMJEguidelines for publication of clinical research and a completedCONSORT checklist must be included with all submissions.

Clinical trial registration

NCT01790698

Study protocol

Described in:
Madsen, M. B. et al. Patient's characteristics and outcomes in necrotising soft-tissue infections: results from a Scandinavian, multicentre, prospective cohort study. Intensive Care Med 45, 1241-1251 (2019).

Madsen, B.B. et al. Necrotizing soft tissue infections - a multicenter, prospective observational study (INFECT): protocol and statistical analysis plan. Acta Anaesthesiological Scandinavia 62, 272-279 (2018).

Data collection

Described in:
Madsen, M. B. et al. Patient's characteristics and outcomes in necrotising soft-tissue infections: results from a Scandinavian, multicentre, prospective cohort study. Intensive Care Med 45, 1241-1251 (2019).

Madsen, B.B. et al. Necrotizing soft tissue infections - a multicenter, prospective observational study (INFECT): protocol and statistical

analysis plan. Acta Anaesthesiological Scandinavia 62, 272-279 (2018).

Outcomes	Described in:<br>Madsen, M. B. et al. Patient's characteristics and outcomes in necrotising soft-tissue infections: results from a Scandinavian, multicentre, prospective cohort study. Intensive Care Med 45, 1241-1251 (2019).<br><br>Madsen, B.B. et al. Necrotizing soft tissue infections - a multicenter, prospective observational study (INFECT): protocol and statistical analysis plan. Acta Anaesthesiological Scandinavia 62, 272-279 (2018).
